# An Aid to Decision-Making in Therapy of Retroperitoneal Fibrosis: Dynamic Enhancement Analysis of Gadolinium MRI

**DOI:** 10.4021/jocmr1254e

**Published:** 2013-01-11

**Authors:** Alexander Sascha Brandt, Lars Kamper, Sonja Kukuk, Werner Piroth, Patrick Haage, Stephan Roth

**Affiliations:** a Departments of Urology, Helios Klinikum Wuppertal, University of Witten/Herdecke, Wuppertal, Germany; b Diagnostic and Interventional Radiology, Helios Klinikum Wuppertal, University of Witten/Herdecke, Wuppertal, Germany

**Keywords:** Retroperitoneal fibrosis, Ormond’s disease

## Abstract

**Background:**

Idiopathic retroperitoneal fibrosis (IRF) as an uncommon cause of obstructive uropathy is often primarily treated medically by the attending urologist. We evaluated dynamic enhancement analysis (DEA) as a possible predictor of response to medical treatment and for treatment monitoring.

**Methods:**

From 2007, 24 patients with fibrosis were assessed by magnetic resonance imaging (MRI) with DEA. The dynamic enhancement quotient (DEQ) was measured before therapy with prednisone (n = 12) or tamoxifen (n = 12) and in follow-up investigations after 3 and 6 months. Response to medical treatment was recorded by changes in the retroperitoneal mass on MRI and possible relief of ureteral obstruction, which was monitored by intravenous pyelogram and/or MAG3 scan after removal of DJ stents.

**Results:**

Treatment groups did not differ significantly as to age, gender, or laboratory values, and response to medical treatment showed no significant difference between agents. Overall there were no cases of progression, 2 cases of stable disease, 11 cases of mild fibrotic regression, and 11 of significant or complete regression. DJ stents could successfully be removed in 21 of 35 renal units (60.0%). In a total of 61 DEAs the DEQ was significantly higher (P < 0.001) in patients with a good response (DEQ = 4.02) than in those with an average response (3.11) or none (2.14).

**Conclusions:**

DEA was able to distinguish between patients with different response rates to medical treatment of IRF and may be useful to individualize therapeutic decision-making.

## Introduction

Retroperitoneal fibrosis as an infrequent cause of obstructive uropathy was first described by Albarran in 1905 [1]. After the first report in English by Ormond in 1948, it became known as a self-contained disease, eponymously styled Ormond’s disease [2].

In more than two-thirds of patients, the cause remains unclear (idiopathic retroperitoneal fibrosis or IRF). In others, fibrosis occurs secondarily, for example, after medical or surgical treatment, infection, neoplasm, trauma, or radiotherapy [3, 4].

The diagnosis of IRF is often established primarily with computed tomography (CT) or magnetic resonance imaging (MRI); typically a retroperitoneal mass is evidenced surrounding the aorta from beneath the renal vessels to the aortic branch. With an atypical formation the diagnosis should be clarified histologically [3, 5].

Traditionally, the relief of urinary tract obstruction has been surgical, but at present the primary approach is often medical after initial relief with ureteral stents [6-8]. The goals of therapy are to remove the ureteral obstruction and to avert progression and recurrence of the fibrosis [3].

Attending physicians have to decide when to abandon medical treatment and perform surgical release of ureteral obstruction, but they can as yet rely only on parameters such as acute-phase reactants whose predictive value of a retroperitoneal mass decrease is uncertain [9].

Burn et al showed in 7 patients at a known disease stage that dynamic gadolinium enhancement in MRI was useful in differentiating newly diagnosed IRF from treated chronic disease [10]. We used this approach in follow-up investigations to study whether it could be used to differentiate between different response rates to medical treatment.

## Patients and Methods

From April 2007 to March 2010, patients who were referred to our department with newly diagnosed IRF were examined with dynamic enhancement analysis (DEA). If need be, renal drainage was done by ureteral stenting; no patient had undergone medical therapy. Demographic, symptomatic, laboratory and radiographic data were recorded in the Else Kroner-Fresenius Registry of Retroperitoneal Fibrosis in Germany, a nationwide registry headquartered in our department [11].

Patients received initial MRI with dynamic gadolinium enhancement. After careful exclusion of malignant disease, medical therapy was begun with either prednisone or tamoxifen according to the contraindications for these agents and patient preference. Prednisone was given at a dose of 1 mg/kg body weight every second day for 10 weeks; thereafter 40 mg/day for 2 weeks, 20 mg/day for 2 weeks, 10 mg/day for 2 weeks, then 5 mg/day. Those receiving tamoxifen took 20 mg twice a day. Medical therapy was given for a total period of one year. All patients gave informed consent to treatment.

In follow-up examinations after 3, 6 and 12 months, response to treatment was evaluated by 3 independent observers (2 radiologists, 1 urologist) who assigned the change in retroperitoneal mass to one of four categories: (0) progression of disease, (I) stable disease, size reduction < 20%; (II) mild regression of fibrosis, reduction 20-50%; (III) significant or complete regression, reduction > 50% or no further delineable fibrosis. Dynamic gadolinium enhancement MRI was repeated in follow-up examinations after 3 and 6 months.

In cases of fibrosis regression and in accordance with the patient’s wish, DJ stents were removed and success was evaluated by intravenous pyelogram and/or MAG3 scan. After 6 and 12 months each case was reevaluated to decide whether to proceed with or change medical therapy or to perform surgery for ureteral obstruction. After successful medical or operative therapy patients were followed-up by MRI twice a year for the first and once a year afterwards.

### Dynamic enhancement analysis in gadolinium-enhanced MRI

MRI was performed with a 1.5-Tesla scanner (Siemens MAGNETOM Avanto, Siemens Medical Systems, Erlangen, Germany) in combination with DEA to evaluate extent and activity of the IRF. Transverse and coronal standard T2-weighted images were acquired before injection of weight-adapted Gadoteridol (ProHance, Altana Pharma, Konstanz, Germany) and T1-weighted images before and after injection.

The T1-weighted DEA was performed in 13 repeated scans at the same table position in defined intervals: 7.5 sec between the first six and 17.5 sec between the last seven.

**Figure 1:**
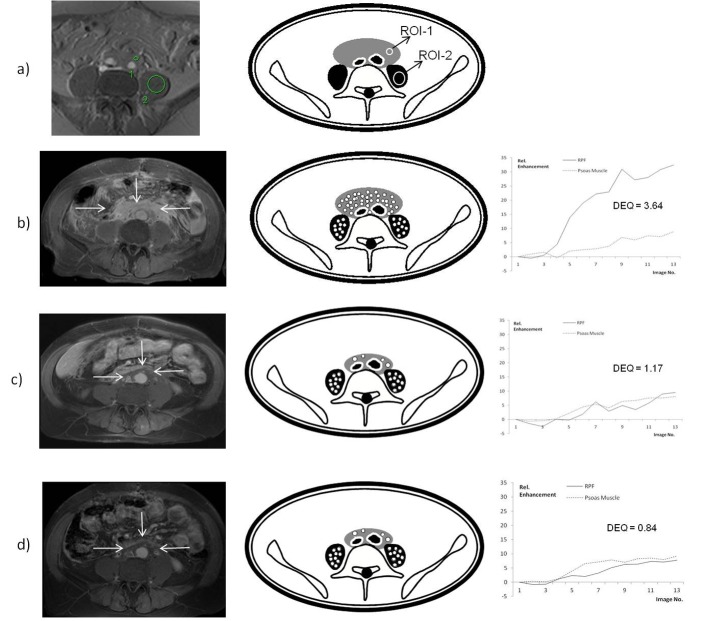
(a) After contrast injection, DEA recorded contrast enhancement of IRF (ROI 1) and psoas muscle (ROI 2), which generates typical curves of the dynamic intensities by the “Mean-Curve” software package. (b) DEA shows a high DEQ in a patient before the start of medical treatment with prednisone. (c) After 3 months the retroperitoneal mass is significantly reduced, as is the DEQ. (d) At 6 months the mass shows no further reduction and the DEQ is changed only slightly.

The dynamic enhancement was assessed in specific regions of interest within the IRF (ROI 1) and psoas muscle (ROI 2) with the “Mean-Curve” software package (Siemens Medical Systems, Erlangen, Germany), which generates curves of the dynamic intensities (Fig. 1a). The dynamic enhancement quotient (DEQ) was calculated after Burn and colleagues [10] by dividing the difference between the maximum enhancement and the pre-contrast intensity within the IRF and the psoas muscle (DEQ = Δ ROI 1 _(IRF)_/Δ ROI 2_(psoas)_).

To prevent the development of nephrogenic systemic fibrosis, renal function was determined by serum creatinine value and estimated glomerular filtration rate (eGFR). Patients with eGFR between 60 - 30 mL/min were informed of their higher risk; no gadolinium-based contrast media were used in patients with an eGFR below 30 mL/min.

### Data storage and statistical analyses

All patients gave written consent to storage and analysis of their personal and disease-related data in the Else Kroner-Fresenius Registry of Retroperitoneal Fibrosis. For data storage we used an SQL database in pseudo-anonymous form, conforming to the standards of the ethics committee of the University Witten/Herdecke.

Statistical analyses were performed with the Wilcoxon rank-sum test for two groups and the Kruskal-Wallis test for more. Fisher’s exact test was used for contingency tables. For all tests P < 0.05 was considered statistically significant. All tests were performed with commercial software (Microsoft Excel®, XLSTAT®).

## Results

A total of 24 patients with newly diagnosed IRF were assessed. Mean age at onset was 54.6 ± 10.8 (37 - 68) years; 4 were women (16.7%) and 20 men (83.3%). Mean follow-up is 41.1 (21 - 61) months. Diagnosis had been secured by histologic proof in 16 patients (CT-guided biopsy in 8, open or laparoscopic biopsy in 8). In the other 8 cases IRF was assumed by the typical presentation on CT imaging. In 24 patients insertion of DJ stents was necessary (12 bilateral, 12 unilateral) at onset of disease.

Twelve patients received initial therapy with prednisone (Group A), 12 with tamoxifen (Group B). There were no statistically significant differences between groups regarding age (P = 0.966), gender (P = 0.590), initial laboratory measurements, and distribution of bi- or unilateral hydronephrosis (Table 1).

**Table 1:** Demographic Data of Patients and Response to Medical Treatment

	Total	Prednisolone	Tamoxifen	P-Value
Number	24	12	12	
Age at onset (years)	54.6 ± 10.8	54.7 ± 9.3	54.6 ± 12.5	0.966
Male	20	11	9	0.590
Female	4	1	3	
ESR after 1 hour (mm)	47.9 ± 31.2	38.0 ± 28.3	57.8 ± 31.9	0.110
ESR after 2 hours (mm)	71.4 ± 29.6	62.6 ± 27.8	80.2 ± 30.0	0.110
CRP (mg/dL)	2.0 ± 2.4	1.5 ± 2.2	2.4 ± 2.6	0.181
Obstructed renal units (n)	34	18	16	0.752
Obstructed renal units (%)	70.8	75.0	0.667	
Initial DEQ	3.5 ± 1.0	3.1 ± 0.8	3.9 ± 1.0	0.100
Progression of fibrosis (after 3/6 months treatment)	0 (0/0)	0 (0/0)	0 (0/0)	0.761
Stable Disease < 20%	2 (3/11)	2 (2/7)	0 (1/4)	(1.00/0.195)
Mild Regression < 50%	11 (10/9)	5 (5/3)	6 (5/6)	
Significant/Complete Regression > 50%	11 (11/4)	5 (5/2)	6 (6/2)	
Successful DJ-stent removal (after 6 months)	23	11	10	0.303
Successful DJ-stent removal (total) (%)	58.8	61.1	62.5	
Final surgical therapy (n)	7	5	2	0.371

After 12 months of therapy there were no cases of disease progression; IRF remained stable in 2, regressed mildly in 11, and significantly or completely in 11. Treatment groups showed no statistically significant difference in response at each follow-up interval and for the total period of medical treatment (Table 2).

**Table 2:** Response to Medical Treatment

	Group A: Prednisolone			Group B: Tamoxifen			Total

	Total	3 months	3 - 6 months	Total	3 months	3 - 6 months	
Category 0:	0	0	0	0	0	0	0
Progression							
Category I:	2	2	7	0	1	4	2
Stable Disease							
Category II:	5	5	3	6	5	6	11
Mild Regression							
Category III:	3	5	0	4	6	0	7
Significant Regression							
Category IV:	2	0	2	2	0	2	4
Complete Regression							
Total		12			12		24

* No statistical significant difference between groups for 1 - 12 weeks (P = 1.00), 12 - 26 (P = 0.195) and after the total period (P = 0.761) of medical treatment.

DJ stents could successfully be removed after 12 months of medical therapy in 15 of 23 patients (65.2%) for a total of 23 of 35 renal units (65.7%). Of these 15 patients, 7 had been on prednisone (11 of 18 renal units; 61.1%) and 8 on tamoxifen (10 of 16 renal units; 62.5%) reaching no statistical significance between groups. In the other 8 patients either renogram or MAG3 scan or both showed ureteral obstruction after stent removal, necessitating reinsertion.

Of these 8 patients one was lost in follow-up and 7 received final operative therapy: ureterolysis in 3, psoas-hitch ureterocystoneostomy in 1 and ureteral reconstruction with ileum segments in 2. In one patient nephrectomy was performed due to loss of function.

In follow-up 2 patients developed recurrent disease after initial successful medical treatment of IRF. Both patients had initial treatment with prednisone. Mean period to relapse was 8.5 (3 and 14) months.

### Dynamic enhancement analysis

Dynamic enhancement analysis (DEA) was evaluated in a total of 61 MRI procedures (Fig. 1): 23 initial examinations, 22 at 3 months and 16 examinations at 6 months. In 1 patient at initial imaging and 1 at follow-up, gadolinium contrast could not be injected owing to a reduced eGFR. In a total of 9 patients follow-up DEA could not be done because the small amount of remaining fibrosis resulted in artifacts of measurement.

**Figure 2:**
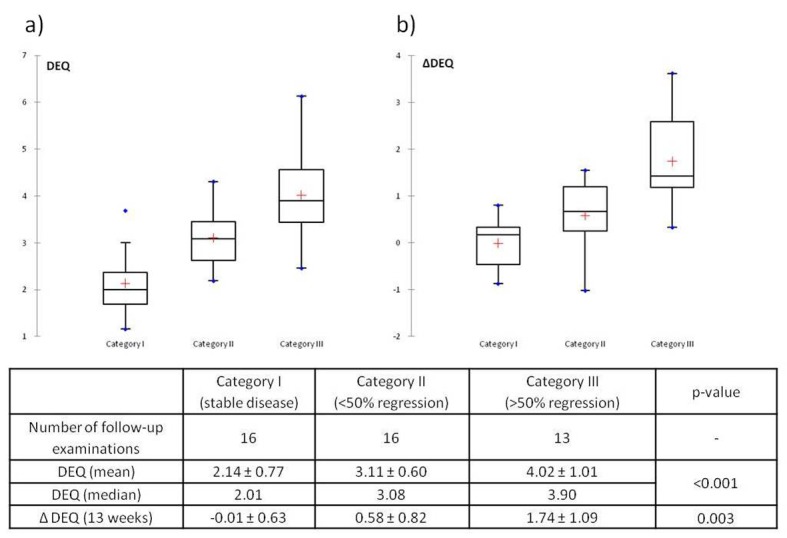
Initial dynamic enhancement quotient (a) and changes in DEQ (b) with reference to subsequent response to medical therapy. Boxplots demonstrate mean values ± S.D., median, maximum and minimum values of DEQ and ΔDEQ.

A total of 16 DEAs were followed by stable disease (Category I), 16 by mild regression (Category II), and 13 by significant or complete regression (Category III). Category I showed the lowest mean DEQ, 2.14 (Fig. 2a). The Kruskall-Wallis test showed a statistically significant difference between groups (P < 0.0001) and the Wilcoxon rank-sum test revealed statistically significant differences of DEQ between each category (I and II, P = 0.007; I and III, P < 0.0001; II and III, P = 0.001).

In Category I (stable disease) there was almost no change in DEQ (ΔDEQ = -0.01 ± 0.63), whereas Categories II and III showed a regression of DEQ after treatment that was highest in Category III (Fig. 2b). The Kruskall-Wallis test again showed statistically significant differences between categories (P = 0.003), and the Wilcoxon rank-sum test showed statistical significance of ΔDEQ between categories I and III (P = 0.001) and II and III (P = 0.013), but not between categories I and II (P = 0.122).

## Discussion

Idiopathic retroperitoneal fibrosis remains a disease rather seldom treated in urologic practice. Thus far the response to medical treatment has been assessable only by clinical tests such as regression of urinary obstruction or reduced size on imaging. Furthermore, there are no parameters able to predict whether medical therapy will be successful. Magrey et al showed that acute-phase reactants such as erythrocyte sedimentation rate and c-reactive protein at baseline are only poor predictors of a therapeutic response to glucocorticoid therapy [9]. For this reason the point at which to abandon medical treatment for surgical intervention is individual and nearly random.

It is reported that investigators typically use medical therapy as long as contrast uptake is evident in the retroperitoneal mass [12]. With DEA we were able to measure and objectify this uptake, whereas in a prior investigation we found that quotients of T1 and T2 signal intensities were not useful [13]. Therefore we think that DEA could be used to determine the right time for surgical intervention and could spare patients disappointing medical therapies with corresponding side effects.

In a series of 7 patients Burn et al showed for the first time that dynamic contrast-enhanced MRI can differentiate between newly diagnosed active fibrosis and treated chronic disease [10]. They based their findings on the different histopathologic appearance of IRF: in its early stage increased vascularity and vessel permeability associated with active inflammation [14] result in a higher local concentration of gadolinium; in later stages predominantly fibrous and collagenous tissue [14] allows a lower concentration.

In accordance with their approach, we used DEA in initial and follow-up examinations of patients receiving medical treatment for IRF. We observed different responses to medical treatment and found the DEQ to be a robust predictor: an above-average response to treatment appears to be associated with a higher DEQ than an average response or none, and differences were significant between groups. Additionally, a better response to medical treatment was associated with a greater decrease in contrast enhancement. In this way DEA, which has been used in other diseases, e.g. to differentiate between benign and malignant soft-tissue lesions [15] and to assess response in breast [16] and prostate cancer [17], could help individualize the medical treatment of IRF.

The advantage of MRI in combination with DEA, especially if multiple examinations are necessary, is that it is not associated with radiation as CT is. Additionally, MRI provides better contrast with surrounding retroperitoneal tissue and does not require iodinated contrast media [5]. A disadvantage is certainly that, because many patients with IRF suffer from renal insufficiency, the risk of gadolinium-associated nephrogenic systemic fibrosis must be carefully considered [18].

Several approaches to medical treatment have been described in the literature, but the lack of controlled trials has meant that treatment has not been standardized and is still largely empirical [7]. Because of the nonspecific inflammatory nature of IRF, corticosteroids are often used at onset [19]. Steroid therapy alone has shown sufficient regression of fibrosis, reported to be over 80% [20-24], but recurrence rates up to 25% have led several authors to propose steroid-sparing agents [25-28].

Tamoxifen has been described as a possible treatment in several anecdotal case reports since 1991 [29]. It seems to have anti-inflammatory or anti-fibroblastic activity in addition to its antiestrogenic effects [7]. Van Bommel et al in 2006 published the first extensive series with tamoxifen monotherapy in 19 patients and showed a slow but steady regression of the mass in 14 of 15 clinical responders with almost no side effects [29].

In our series we found regression of fibrosis in 22 of 24 patients (91.7%) after 6 months’ treatment, with no statistical significance between prednisone and tamoxifen (P = 0.761). Even though regression occurred in 91.7%, the ureteral stent could only be removed in 65.7% of renal units after 12 months. The other patients needed final surgical treatment.

Our series is limited by the small number of patients. Furthermore, patients were not randomized to therapy; their preference influenced the agent used. Therefore, further studies of treatment outcome with more patients and the presentation of long-term results are planned.

The evaluation with DEA is limited in patients with very small paravascular fibrotic plaques or with significant fibrotic regression because of restricted space for the manual positioning of ROIs. False results may occur if the ROI includes intravascular contrast enhancement owing to aortic pulsation, which led to exclusion of 9 patients in our series. Inhomogeneous contrast enhancement in different parts of the IRF may also limit the assessment of therapeutic response.

Upcoming studies must validate the method presented here in long-term follow-up to determine whether the DEQ can prevail as a safe and reliable predictor of therapeutic success.

### Conclusion

Dynamic enhancement analysis was able to distinguish between patients with different response rates to medical treatment of retroperitoneal fibrosis. It appears that assessment of the dynamic enhancement quotient could be used to individualize medical treatment and to provide additional information for therapeutic decision-making: whether to continue medical therapy or to perform final surgical relief of ureteral obstruction. Additionally, therapy monitoring is possible for different therapeutic agents with DEA. Further investigations are mandatory to confirm that the DEQ can be a reliable predictor of response to medical treatment.
